# Impaired l-arginine metabolism marks endothelial dysfunction in CD73-deficient mice

**DOI:** 10.1007/s11010-019-03537-4

**Published:** 2019-05-15

**Authors:** P. Mierzejewska, M. A. Zabielska, B. Kutryb-Zajac, M. Tomczyk, P. Koszalka, R. T. Smolenski, E. M. Slominska

**Affiliations:** 1 Department of Biochemistry, Medical University of Gdansk, Debinki 1, 80-211 Gdańsk, Poland; 2 Department of Physiology, Medical University of Gdansk, Gdańsk, Poland; 3 Department of Medical Biotechnology, Intercollegiate Faculty of Biotechnology UG-MUG, Medical University of Gdansk, Gdańsk, Poland

**Keywords:** Endothelium, Endothelial dysfunction, Nucleotide metabolism, Ecto-5′-nucleotidase, Adenosine, l-Arginine metabolism

## Abstract

Changes in the ecto-5′-nucleotidase activity—an extracellular nucleotide catabolic enzyme may lead to the inflammation and endothelial dysfunction. We investigated the effect of CD73 deletion on the endothelial function and l-arginine metabolism in various age groups of mice. 1-,3-,6-, and 12-month-old, male C57BL/6 J wild type (WT) and C57BL/6 J CD73−/− (CD73−/−) mice were used. Blood samples were used for the analysis of adenine nucleotide concentrations. Serum samples were analyzed for the concentration of amino acids, Interleukin 6 (IL-6), Intercellular Adhesion Molecule 1 (ICAM-1), Vascular Cell Adhesion Molecule 1 (VCAM-1), and endothelial nitric oxide synthase (eNOS) level. Serum and aortic nitrate/nitrite, as well as aortic arginase and NOS activity in endothelial cells (EC) were evaluated. CD73 deletion led to age-dependent increase in IL-6, ICAM-1, and VCAM-1 concentration compared to WT. All CD73−/− mice age groups were characterized by reduced l-Arginine concentration and eNOS level. Significantly lower NOS activity was noticed in EC isolated from CD73−/− mice lungs in comparison to EC isolated from WT lungs. The l-Arginine/ADMA ratio in the CD73−/− decreased in age-dependent manner in comparison to WT. The nitrate/nitrite ratio was reduced in serum and in aortas of 6-month-old CD73−/− mice as compared to WT. The ornithine/arginine and ornithine/citrulline ratios were increased in CD73−/− compared to controls. Blood (erythrocyte) Adenosine-5′-triphosphate and Adenosine-5′-diphosphate levels were reduced in favor to higher blood Adenosine-5′-monophosphate concentration in CD73−/− mice in comparison to WT. The CD73 deletion leads to the development of age-dependent endothelial dysfunction in mice, associated with impaired l-arginine metabolism. CD73 activity seems to protect endothelium.

**Electronic supplementary material:**

The online version of this article (10.1007/s11010-019-03537-4) contains supplementary material, which is available to authorized users.

## Introduction

Nucleotides and their catabolites play a significant role in the cell energetics and regulation, where they act through both, intracellular and extracellular mechanisms. In the extracellular space, they are important regulators of the inflammation and immune response [1]. Adenine nucleotides and nucleosides are degraded by cell surface ecto-enzymes. One of the most important is ecto-5′-nucleotidase, also known as CD73. It catalyzes the hydrolysis of the adenosine-5′-monophosphate (AMP) to adenosine [2]. CD73 has a wide tissue distribution. It is present in kidney, liver, lung, brain, and heart, as well as on leukocytes, thymus, spleen, and lymph nodes. Such widespread CD73 distribution is related to its high abundance in endothelium [3]. The adenosine, produced by CD73, is considered a molecule with an important signaling role, which is mediated by the activation of the P1 receptors. The decrease in the extracellular adenosine concentration may lead to the development of the inflammation and endothelial dysfunction [4].

Endothelial dysfunction is involved in the pathophysiology of all cardiovascular diseases. It is manifested by the increased permeability of endothelial cells, elevated adhesion molecules expression, intensified cytokines and chemokines secretion, leukocytes adhesion, platelet activation, and also facilitation of migration and proliferation of vascular smooth muscle cells [5]. The mechanisms underlying this disorder are related to an active inflammatory process and reduced bioavailability of nitric oxide (NO). NO is mainly formed in endothelial cells from l-arginine by endothelial NO synthase (eNOS) [6].

Our earlier studies demonstrated the strong association between the lack of the CD73 activity and the development of the aortic valve dysfunction in mice [7]. It is also known, that binding of the lymphocytes to the endothelium masks endothelial CD73 without any covalent modification [8]. Reduced level of extracellular adenosine on lymphocyte that contact with endothelial cells (EC) leads to an increased permeability of EC that contributes to enhanced leukocyte transmigration [9, 10]. Other literature data also confirmed the involvement of CD73 in modulation of coronary vascular tone and platelet activation, as well as inhibition of leukocyte adhesion [11]. This study aimed to investigate the effect of the CD73 deletion on the endothelial function in mice at various age and relate it to l-arginine-dependent signaling pathways.

## Materials and methods

### Animals maintenance

All experiments were conducted in accordance with a Guide for the Care and Use of Laboratory Animals published by the European Parliament, Directive 2010/63/EU and were performed with approval of the Local Ethical Committee for Animal Experimentation in Bydgoszcz (27/2016). C57BL/6 J CD73−/− (CD73−/−) mice were obtained from Heinrich-Heine-Universität in Düsseldorf, Germany [11]. C57BL/6 J Wild Type (WT) (*n* = 27) and CD73−/− (*n *= 27) mice, fed standard chow diet, were used for the experiments. At the age of 1, 3, 6, and 12 months, mice were subjected to anesthesia—ketamine (100 mg/kg) and xylazine (10 mg/kg). Blood and serum samples were collected and immediately frozen in liquid nitrogen. Blood samples were collected through the tail vein puncture of live mice. Following tail vein puncture, blood dripped directly into the Eppendorf tube previously cooled in liquid nitrogen, after which the bleeding was stopped with the gauze. Serum samples were obtained from peripheral blood after centrifugation (×1700 g, 10 min, 21 °C). Then, after opening the chest, the thoracic and abdominal aortas were removed, placed in physiological saline and dissected from the surrounding tissues.

### Determination of amino acids concentration

To evaluate the concentration of plasma amino acids, as well as l-arginine analogs, an aliquot of serum (50 µl) was extracted with acetonitrile (ratio 1:2.4) and centrifuged (×20,800 g/10 min/4 °C). Supernatant were collected and freeze dried. The precipitate was dissolved in water at a volume equal to the initial plasma volume. Amino acids and derivatives concentration were determined using high performance liquid chromatography–mass spectrometry (LC/MS). The system contained a Surveyor MS autosampler, quaternary Surveyor MS pump, and a degasser connected to TSQ Vantage triple quadrupole mass detector. Heated electrospray ionization in positive mode was used. The column used for separation was a 50 × 2 mm Synergi Hydro-RP 100 with a particle size of 2.5 µm. Mobile phase composed of water with 5 mM nonafluoropentanoic acid (A) and acetonitrile with 0.1% formic acid (B). Mobile phase was delivered at 0.2 ml/min and volume of injection was 2 µl. The identity of individual amino acids and internal standard 2-chloroadenosine was confirmed by the similarity of molecular weights, fragmentation pattern, and chromatographic retention time [12].

Intracellular adhesion molecule-1 (ICAM-1), vascular adhesion molecule-1 (VCAM-1), interleukin 6 (IL-6), and tumor necrosis factor-alpha (TNF-alpha) concentration, as well as endothelial nitric oxide synthase level (eNOS) measurements.

The soluble ICAM-1, VCAM-1, and IL-6 serum levels (Sigma-Aldrich, RAB0220, RAB0506, RAB0308, RAB0477), as well as eNOS content (Wuhan EIAan Science, E0815 m) were determined using enzyme-linked immunosorbent assay kits according to the manufacturer’s protocols.

### Isolation of murine lung endothelial cells

For isolation, five CD73−/− and eight WT mice at the age of about 8 weeks were used for our procedure. Mice were anesthetized with ketamine (140 mg/kg) and xylazine (14 mg/kg) followed by opening the chest. Murine lung tissues were harvested, placed into Petri dishes containing Dullbecco’s Modified Eagle’s Medium (DMEM) with low glucose supplemented with penicillin–streptomycin (Sigma-Aldrich), and cut into small pieces. The minced tissue and DMEM were collected in a 15 ml tube and centrifuged (234 g, rt, 5 min) to remove erythrocytes and blood plasma. Supernatant has been removed. Tissue pellet was washed with supplemented DMEM and centrifuged again (under the conditions mentioned above). Then, the tissue pellet was suspended in Collagenase Type A solution (2.5 mg/ml in a DMEM low glucose, Gibco) mixed and incubated at 37 °C for 60 min. After digestion with collagenase, the 70 µm cell strainer was used to get the single cell suspension. The cell pellet was washed in Dulbecco’s Phosphate-Buffered Saline (DPBS) and centrifuged twice (234 g, rt, 5 min). After final centrifugation, a supernatant was removed and cells were resuspended in DMEM with d-Valine (glucose 4.5 g / l (Immuniq), 10% FBS, Endothelial cell growth supplement—ECGS 15 mg/500 ml, 2 mM l-glutamine and penicillin–streptmycin) and plated into a T-25 tissue culture flask. The next day, the medium containing suspended and weakly adherent cells was transferred to a new culture flask. For the experiments cells between two to five passage were used.

### Measurement of nitric oxide synthase activity in murine endothelial cells

For determination of endothelial nitric oxide synthase activity, CD73−/− and WT lung endothelial cells were cultured in 96 well plates at a density 100,000 cells per well. After 24 h, nitric oxide production from murine EC was assessed using spectrofluorimeter Niric Oxide Synthase Detection System (Sigma-Aldrich, FCANOS1), according to the manufacturer’s protocol. For fluorometric NO measurement, the cell-permeable NOS-derived NO—sensitive 4,5-diaminofluorescein diacetate (DAF-2DA) was used. Diphenyleneiodonium chloride (DPI) was used as an ihibitor of inducible NOS (iNOS) at a concentration of 1 uM (as recommended in the protocol). The results are shown as relative fluorescence units (RFU) of NO concentration per mg of protein.

### Determination of serum and aortic nitrate/nitrite levels

Serum nitrate/nitrite concentrations were determined using the nitrite/nitrate assay kit (Sigma-Aldrich, 23479) according to the manufacturer’s protocol. NO metabolites concentration was measured by the Griess reaction, in which NO_3_^−^ is converted to NO_2_^−^ by the nitrate reductase. Following the enzymatic reaction, the Griess reagent was added to the samples. The absorbance of the azo compound was measured spectrophotometrically at a wavelength of 540 nm. The azo coupling between diazonium species, which are produced from sulfanilamide with NO_2_, and naphthylethylenediamine resulted in a colorimetric product proportional to the NO metabolite present.

CD73−/− and WT mice aortic fragments for the nitrate/nitrite concentration measurement were homogenized in phosphate buffer (pH 7.4) containing Tris-HCl (50 mM), EDTA (0.1 mM), phenylmethylsulphonyl fluoride (0.1 mmol/l), dithiothreitol (0.5 mmol/l), trypsin inhibitor (10 µg/ml), and leupeptin (10 µg/ml). The homogenates were centrifuged (×20,000 g, 4 °C, 15 min) and the supernatants were collected. The total nitrate/nitrite ratio was determined as mentioned above. NO metabolites levels are shown in nmol/mg of tissue [13].

### Measurement of arginase activity

To evaluate arginase activity in aortic fragments of CD73−/− and WT mice, the Arginase Activity Assay Kit (Sigma-Aldrich, MAK112) was used according to the manufacturer’s protocol. Aortas were homogenized in 50 mM Tris-HCl pH 9, centrifuged (×5000 g, 4 °C, 5 min) and the supernatant was rebuffed using a 10 kDa Molecular Weight Cut-Off Filter [14]. The arginase activity is shown as units per g of tissue. One unit of arginase is the amount of enzyme that will convert 1 µmol of l-arginine to ornithine and urea per minute at pH 9.0 in 37 °C.

### Determination of blood nucleotides and metabolites concentration

To determine nucleotides and their metabolites concentration, the whole blood samples were immediately frozen in a liquid nitrogen, extracted with 1.3 M HClO4 (ratio 1:1) followed by a centrifugation (×20,800 g/15 min/4 °C). Supernatants were then collected and brought to pH 6.0–6.5 using 3 M K3PO4 solution. After 15-min incubation on ice, samples were centrifuged again (×20,800 g/15 min/4 °C), and the supernatants were analyzed using high performance liquid chromatography (HPLC) as previously described [15]. Blood AMP hydrolysis rate was determined as described in the Supporting Informations.

### Statistical analysis

The results were presented as mean ± SEM. The statistical analysis was performed using Graph Pad Prism 7 (Graph Pad Software). Unpaired Student *t* test was used for comparisons between two groups. Two-way analysis of variance (ANOVA) followed by Tukey’s Multiple Comparison Test was used to compare more than two groups. A *p* value < 0.05 was considered a significant difference.

## Results

**Fig. 1:**
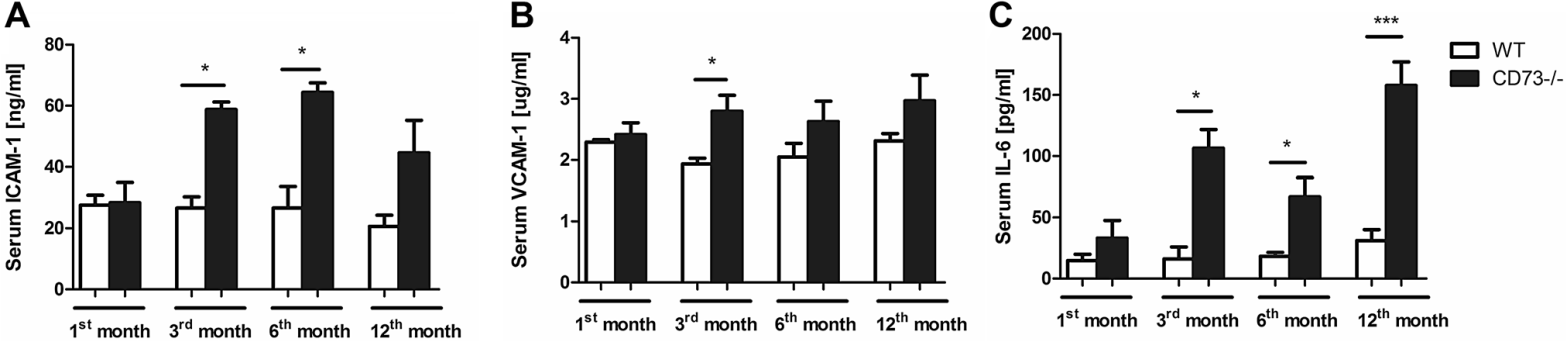
Increase in IL-6 and adhesion molecules concentrations as a suggestion of an endothelium activation and pro-inflammatory phenotype of CD73−/− mice. Serum **a** ICAM-1; **b** VCAM-1 and **c** IL-6 concentration in 1-, 3-, 6-, and 12- month—old CD73−/− and WT mice. All values are shown as mean ± SEM (*n *= 5; Two-way ANOVA with post-hoc Tukey test and Student *t* test: *p

To assess the development of inflammation and confirm the pro-inflammatory phenotype of the studied animals, the concentrations of ICAM-1, VCAM-1, and IL-6 were measured in the 1-, 3-, 6-, and 12-month-old CD73−/− and WT mice serum. The level of both adhesion molecules, associated with the endothelium activation, as well as IL-6 were increased in CD73−/− mice serum as compared to WT, irresprecitive of their age (Fig. 1). The highest concentration of ICAM-1 was observed in 3- and 6-month-old mutants (Fig. 1a). The most increased IL-6 level was noticed in the 12-month-old-CD73−/− mice serum. Additionaly, the concentration of another pro-inflammatory factor—TNF-alpha was measured in the 6-month-old CD73−/− and WT mice serum. The level of this cytokine was approximately three times higher in mutant mice serum in comparison to control group (Supplementary Fig. S1).

**Fig. 2:**
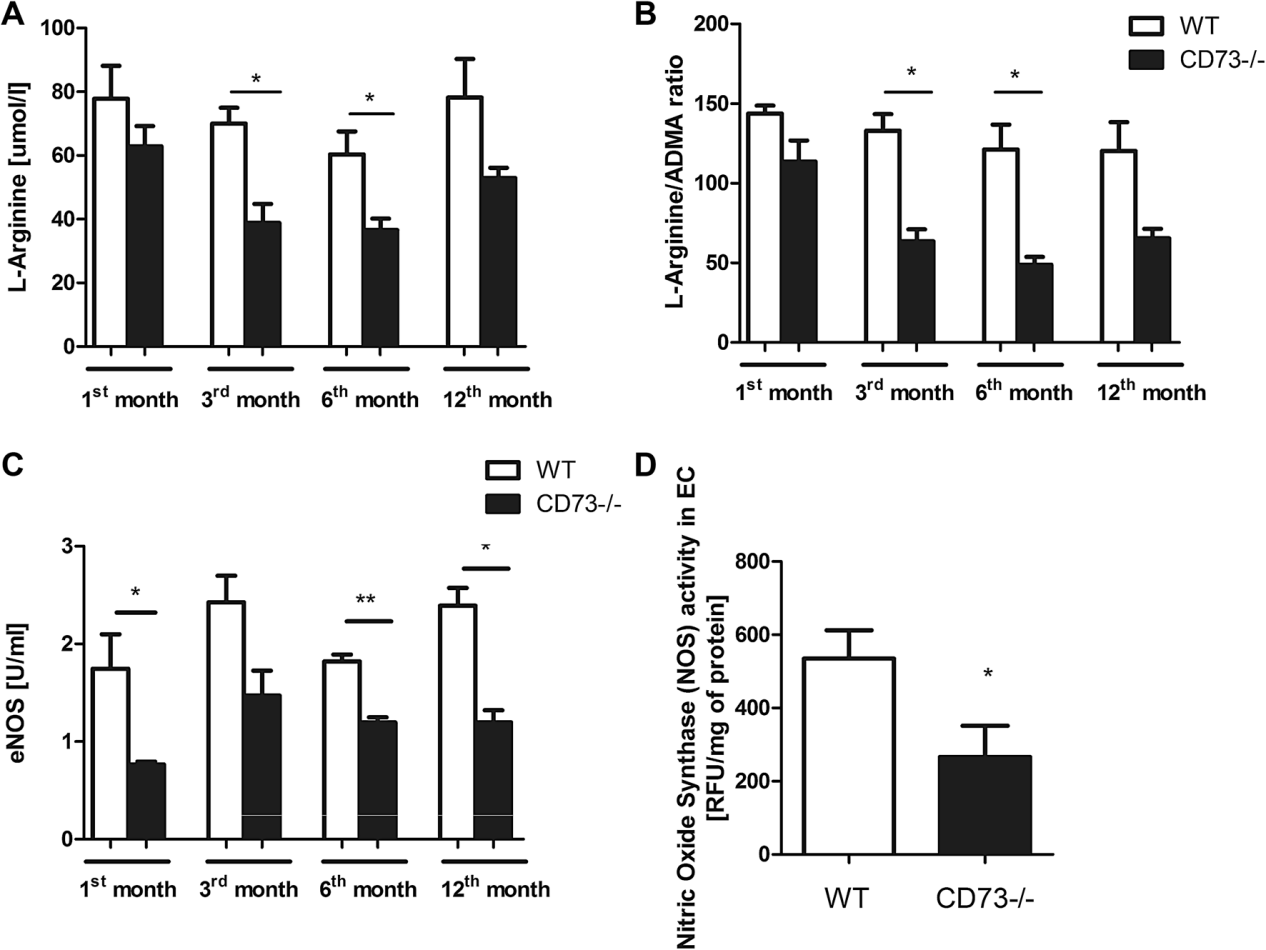
Lower serum l-Arginine level and NOS activity in endothelial cells indicating endothelium dysfunction of CD73−/− mice. Serum **a** Arginine concentration, **b** Arginine/ADMA ratio, and **c** eNOS level of 1-, 3-, 6- and 12- month—old CD73−/− and WT mice. Values are shown as mean ± SEM (*n* = 5; Two-way ANOVA with post-hoc Tukey test and Student *t* test: *p < 0.05; **p < 0.01; ***p < 0.001) **d** NOS activity in EC isolated from 2- month—old CD73−/− and WT mice lungs. Values are shown as mean ± SEM (*n *= 3—in each activity assay 5 wells for each group tested; Student t test: *p < 0.05; **p < 0.01; ***p < 0.001)

The analysis of serum l-Arginine concentration, l-Arginine to asymmetric dimethyl arginine (ADMA) ratio and eNOS level, as well as NOS activity in the endothelial cells isolated from CD73−/− and WT mice are consistent with the development of endothelial dysfunction in mutant mice (Fig. 2). CD73−/− mice were characterized by significantly decreased l-Arginine concentration and considerably lower eNOS level in the serum, regardless of their age (Fig. 2a, c). Moreover, the decrease in the l-Arginine to ADMA ratio, which is strongly related to endothelial dysfunction, was proportional to the age of CD73−/− mice (Fig. 2b). Consistent with the lower eNOS level in serum, the activity of eNOS was significantly reduced in the endothelial cells isolated from CD73−/− mice as compared to WT (Fig. 2d).

**Fig. 3:**
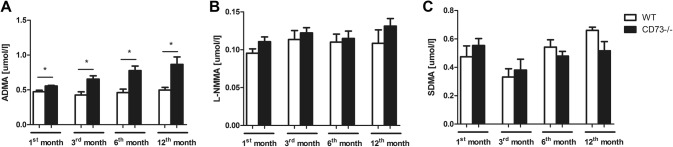
The age-dependent ADMA increase in CD73−/− mice serum. Serum l-arginine analogs: **a** ADMA; **b**l-NMMA and **c** SDMA concentration of 1-, 3-, 6- and 12- month—old CD73−/− and WT mice. All values are shown as mean ± SEM (*n *= 5; Two-way ANOVA with post-hoc Tukey test and Student *t* test: *p < 0.05; **p < 0.01; ***p < 0.001)

We also investigated the l-Arginine analogs concentration: ADMA, l-NG-monomethyl arginine (l-NMMA) and symmetric dimethylarginine (SDMA) in the 1-, 3-, 6- and 12-month-old CD73−/− and WT mice serum. The age-dependent ADMA increase in CD73−/− mice was noticed, as compared to WT (Fig. 3a). There were no significant differences between the l-NMMA and SDMA concentration in control and mutant mice serum, irrespective of their age (Fig. 3b, c).

**Fig. 4:**
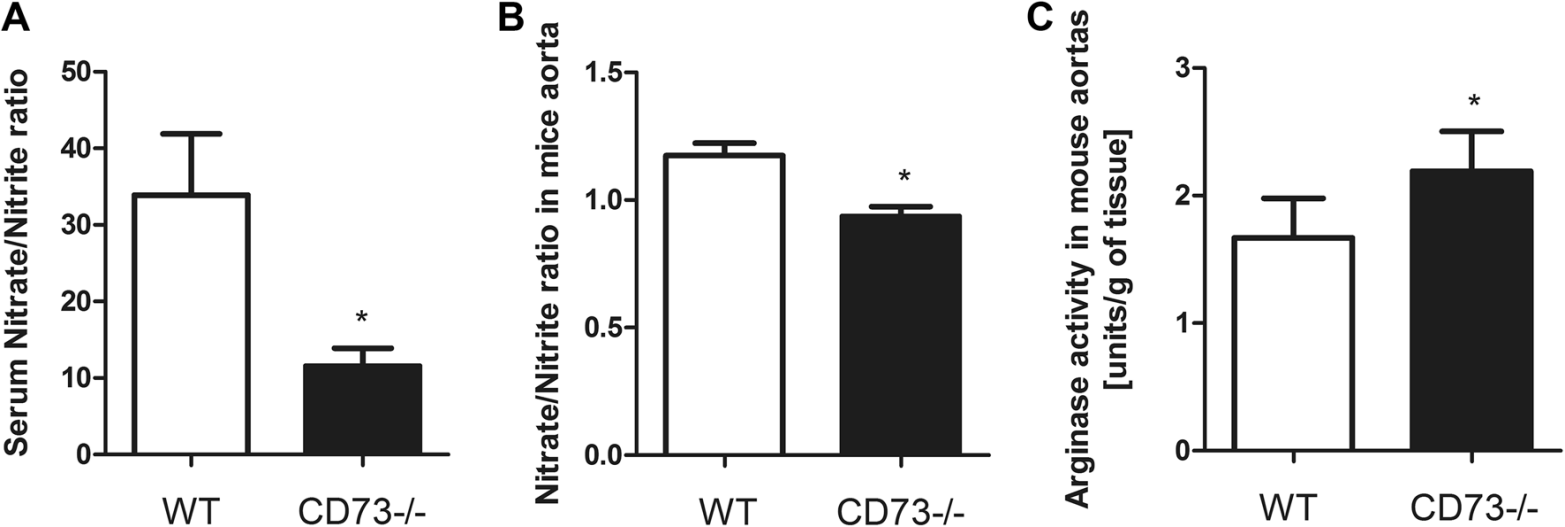
Impaired nitric oxide metabolism in CD73−/− mice, indicated by the reduced serum and aortic nitrate/nitrite ratio. **a** Serum and **b** aortic nitrate/nitrite ratio, as well as **c** aortic arginase activity of 6—month—old CD73−/− and WT mice. All values are shown as mean ± SEM (*n *= 7; Two-way ANOVA with post-hoc Tukey test and Student *t* test: *p < 0.05; **p < 0.01; ***p < 0.001)

To further characterize the implications of a lack of CD73 activity for the l-Arginine metabolism, we evaluated serum and aortic nitrate/nitrite levels, as well as arginase activity in the 6-month-old CD73−/− and WT mice aortas. The nitrate/nitrite ratio was markedly decreased in serum, but also in the aortic fragments of CD73−/− mice as compared to WT (Fig. 4a, b). In addition, we noticed considerably higher activity of arginase in the CD73−/− mice aortas in comparison with controls (Fig. 4c).

**Fig. 5:**
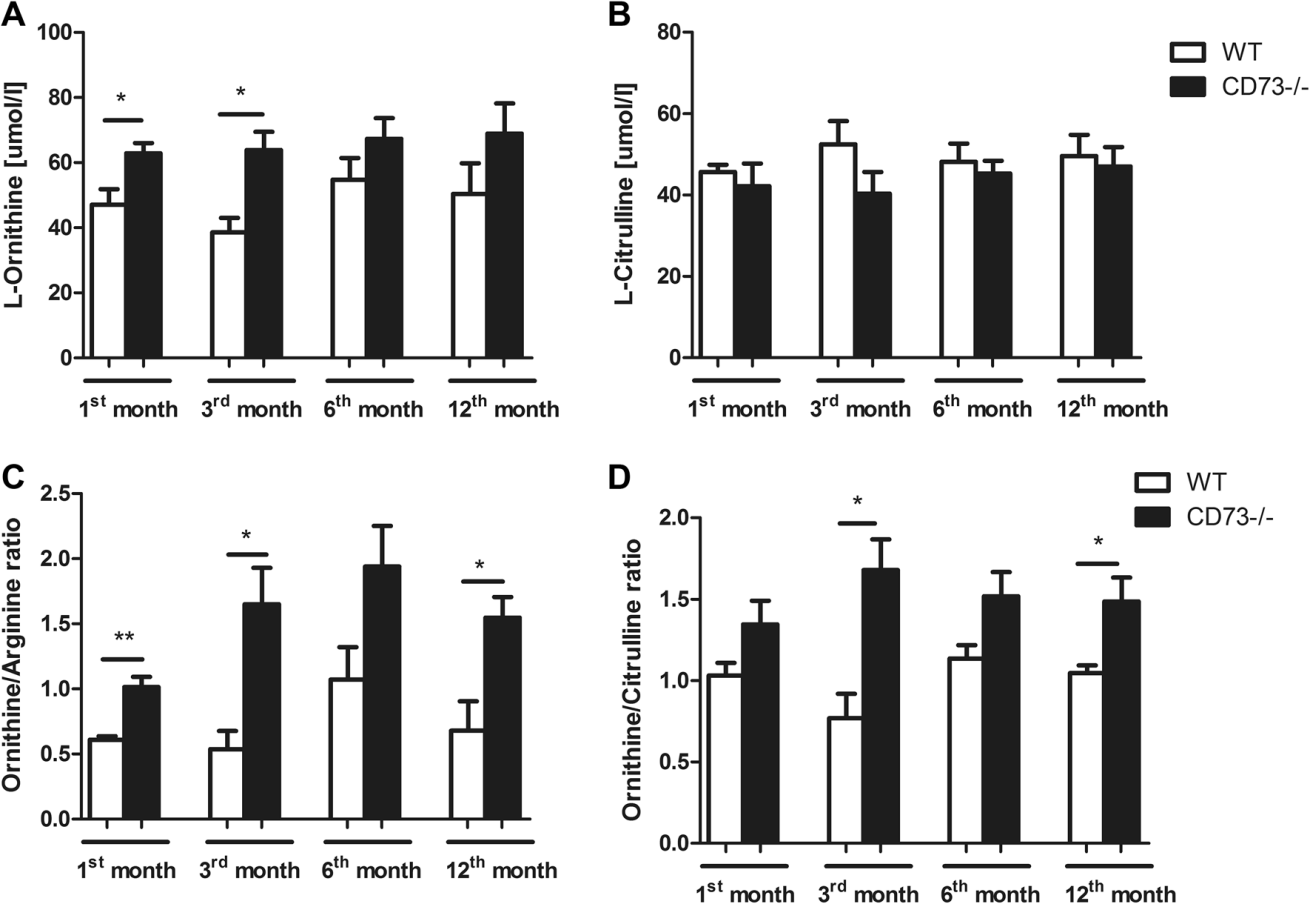
Increased l-Ornithine/ l-Arginine and l-Ornithine/l-Citruline ratio in CD73−/− mice serum. **a** Ornithine and **b** Citruline, as well as **c** Ornithine/Arginine and **d** Ornithine/Citrulline ratios in 1-, 3-, 6-, and 12- month – old CD73−/− and WT mice serum. All values are shown as mean ± SEM (*n *= 5; Two-way ANOVA with post-hoc Tukey test and Student *t* test: *p < 0.05; **p < 0.01; ***p < 0.001)

In the next step, we evaluated the l-Arginine metabolites concentration. We observed the significant increase in ornithine level in the CD73−/− in comparison to control, in particular in 1- and 3-month-old animals (Fig. 5a). There was no difference in citrulline concentration between study groups. Age did not affect changes in the l-Arginine metabolites in the serum of the mice involved in the experiment (CD73−/− and WT) (Fig. 5a, b). Moreover, we observed significantly increased ratios of ornithine/arginine and ornithine/citrulline in all CD73−/− age groups in comparison with controls (Fig. 5c, d).

**Fig. 6:**
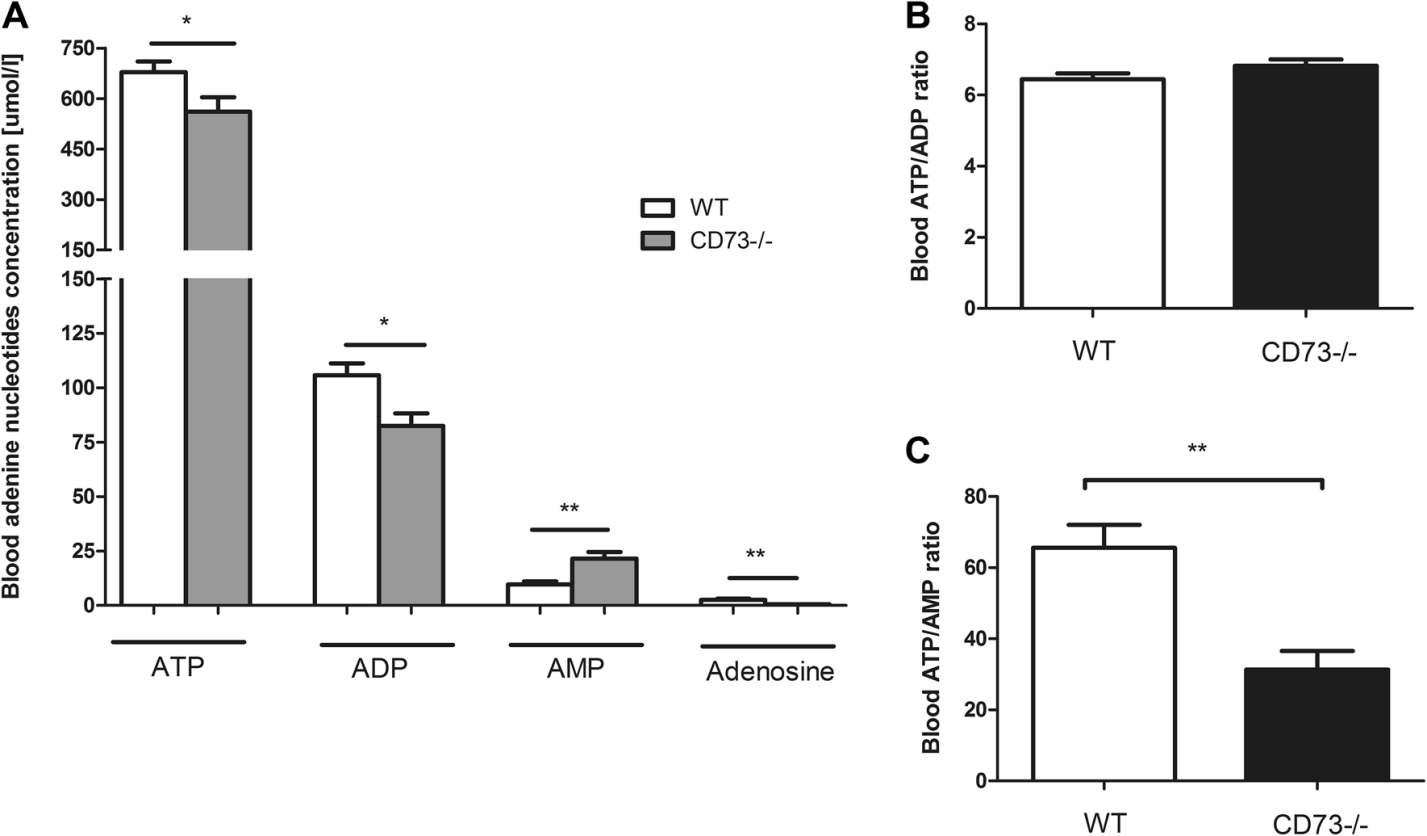
Impaired adenine nucleotides pattern in CD73-deficient mice. **a** Blood adenine nucleotides and adenosine level, **b** blood ATP/ADP ratio and **c** blood ATP/AMP ratio in 6-month-old CD73−/− and WT mice blood. All values are shown as mean ± SEM (*n* = 5; Student *t* test: *p < 0.05; **p < 0.01; ***p < 0.001)

Additionally, we measured the adenine nucleotides and metabolites concentration in whole blood samples of 6-month-old CD73−/− and WT mice. CD73-mutants were characterized by decreased blood ATP and ADP levels and—on the other hand—significantly higher blood concentration of AMP as compared to WT. The adenosine level was considerably lower in CD73−/− mice in comparison to WT (Fig. 6a), which was consistent with deletion of the CD73 encoding gene. The ATP/ADP ratio didn’t differ between both study groups; however, ATP/AMP ratio was significantly lower in CD73-deficient animals (Fig. 6b, c). Furthermore, we observed the significantly reduced AMP hydrolysis rate in mutant mice blood (Supplementary Fig. S2).

Moreover, we also analyzed the other amino acids concentration in 6-month-old CD73−/− and WT mice serum. We noticed some disturbances in the amino acid levels. CD73−/− mice were characterized by elevated concentration of—in particular—alanine, aspartic acid, glutamic acid, as well as lower concentration of tryptophan as compared to WT (Supplementary Table 1).

## Discussion

Our results support the concept of crucial role of the ecto-5′-nucleotidase activity in endothelium homeostasis. The lack of this activity in CD73−/− mice results in vascular endothelial dysfunction, proved by enhanced adhesion molecules and pro-inflammatory cytokine concentration. This study for the first time links the impaired l-Arginine metabolism with endothelial damage in CD73-deficient mice. Changes in the analyzed parameters were progressing with age of animals.

The CD73 gene deletion resulted in significant increase in both adhesion molecules associated with endothelium activation—intracellular adhesion molecule 1 (ICAM-1) and vascular adhesion molecule 1 (VCAM-1), as well as considerably elevated concentration of the pro-inflammatory cytokines—interleukin 6 (IL-6) (Fig. 1) and tumor necrosis factor—alpha (TNF-alpha) (Fig. S2). This pro-inflammatory phenotype of CD73−/− mice was intensified with their age (Fig. 1). CD73 is highly expressed on vascular endothelial cells. Usually, endothelial cells (EC) are resistant to leukocyte adhesion. However, an active inflammatory process can stimulate adhesion molecules—ICAM-1 and VCAM-1 expression by EC, which results in the binding of leukocytes to the vessel wall and their transmigration into peripheral tissue [16]. Binding of leukocytes to EC results in an inhibition of CD73 activity [17]. Other studies also indicated that the monocytes adhesion in ex vivo-perfused carotid arteries of CD73−/− mice has been significantly increased and this pro-inflammatory effect was mediated by the VCAM-1 upregulation [18]. Colgan et al. demonstrated that CD73 deficiency had effect on the endothelial permeability. They observed exacerbation of hypoxia-induced vascular leak in various organs, such as lung, heart, or kidneys, in response to the lack of CD73 activity. Furthermore, a perivascular interstitial edema with inflammatory infiltrates surrounding the larger vessels of the pulmonary vasculature was noticed in CD73−/− mice subjected to hypoxia. This enhanced endothelial permeability may be related to the effect of the A2B receptor [19]. Activation of the A2A and A2B adenosine receptors on neutrophil surface contributes to an anti-adhesive signal and thus decreases binding of neutrophils to endothelial cells. In addition, adenosine significantly diminishes release of the cytokines from the vasculature and leukocytes and therefore inhibits immune response and leukocyte extravasation [20]. All of these findings suggest that CD73 fulfills an important endothelial barrier function. Moreover, in vitro coculture experiments with EC and T lymphocytes demonstrated that genetic deletion of CD73 promotes the transendothelial migration of T lymphocytes and increases the expression of TNF-alpha, VCAM-1, and IFN-Y [21]. In addition, Burghoff et al. demonstrated that CD73−/− mice were characterized by significantly less weight gain and a lower content of white adipose tissue, as well as increased free fatty acids (FFAs) and triglycerides in the serum. High levels of FFAs lead to the reactive oxygen species generation, as well as to the activation of TNF-alpha and IL-6 via the IKK/NF-KB inflammatory signaling and thus contribute to the endothelium cells damage [22, 23].

One of the possible consequences of endothelial dysfunction is the impairment in l-arginine metabolism and therefore decreased vascular nitric oxide (NO) activity. In the EC, NO is produced from l-arginine via the endothelial nitric oxide synthase (eNOS). NO is a key regulator of vasodilation and endothelium homeostasis. Moreover, it plays an important role in regulation of macrophage-induced cytotoxicity and platelet aggregation [24]. Since l-arginine is the only substrate for the eNOS-catalyzed reaction, its availability is criticial in controlling NO production [25, 26]. We observed considerably decreased l-arginine (Fig. 2a) and eNOS serum levels in all age groups of CD73−/− mice (Fig. 2c). Moreover, we found markedly lower NOS activity in EC isolated from CD73−/− mice lungs as compared to EC isolated from WT mice lungs (Fig. 2d), which was accompanied by reduced serum, but also aortic nitrate/nitrite ratio in CD73−/− group in comparison to control (Fig. 4a, b). Impaired production of NO leads to vasoconstriction, leukocyte adhesion and oxidative stress. Oxidative stress itself can also disrupt the production and activity of NO via its inactivation by free radicals and reduction of the tetrahydrobiopterin availability, which is a cofactor required for NO synthesis [27]. Our earlier data indicated significantly lowered plasma total antioxidant status in CD73−/− mice, which may suggest an increase production of free radicals [7], since oxygen radicals are scavenged by CD73-derived adenosine [28]. In addition, Saura M. et al found the association between increased IL-6 concentration and reduction of the eNOS expression in endothelial cells via activation of the signal transducer and transactivator-2 (Stat-3) [29]. It has been also demonstrated that IL-6 may increase the binding between eNOS and caveolin-1, a caveolae scaffold protein that inhibits eNOS [30]. Furthermore, in addition to decreased serum l-arginine concentration, we noticed an elevated asymmetric dimethylarginine (ADMA) serum level (Fig. 3a) in all age groups of CD73−/− mice, what resulted in age-dependent l-Arginine/ADMA ratio reduction (Fig. 2b). Dimethylarginines—ADMA, NG‐mono‐methyl‐l‐arginine (l-NMMA) and NG‐N′G‐dimethyl‐l‐arginine (SDMA) are the products of the methylated proteins degradation. S-adenosylmethionine is a donor of the methyl groups, changing to S-adenosylhomocysteine (SAH) [31]. The formation of dimethylarginines is catalyzed by protein arginine methyltransferase type 1 and 2 (PRMT1, PRMT2). PRMT-1 is responsible for the LNMMA and ADMA formation, while PRMT-2 methylates proteins to release SDMA and l-NMMA. ADMA is an endogenous inhibitor of endothelial nitric oxide synthase (eNOS) that competes with its substrate l-arginine, impairing nitric oxide (NO) production and leading to endothelial dysfunction [32, 33]. Plasma ADMA has been found to be increased in patients with vascular pathologies [34]. Boger et al. found that ADMA concentration was better correlated with endothelial dysfunction than LDL cholesterol in hypercholesterolemic individuals and this increase in ADMA concentration was reversed by l-arginine administration [35]. Considering these data, our results are highly suggestive of age-dependent endothelial dysfunction in CD73−/− mice, leading to l-arginine metabolism disturbances.

We didn’t find significant differences in other l-Arginine analogs—l-NMMA and SDMA—serum levels between CD73−/− and WT mice (Fig. 3). In addition to ADMA, l-arginine can also be methylated to produce SDMA and l-NMMA. ADMA and l-NMMA are competitive inhibitors of eNOS but SDMA does not appear to directly affect eNOS activity. However, all three methylarginines are able to interfere with l-arginine transport to EC, what results in cellular l-arginine deficiency [36]. l-NMMA is as potent as ADMA in eNOS inhibition, but its plasma concentration is approximately 10 times lower. Thus, ADMA seems to be a key player in eNOS activity reduction [32]. On the other hand, SDMA is considered to have a role in renal pathologies. The work of Zoccali et al. described the evaluation of ADMA concentration at the clinical outset of acute inflammation. Acute inflammation and accompanying nitrosative stress was characterized by an increase in the plasma level of ADMA and this phenomenon is accentuated in patients with relatively lower blood pressure. In this study, in contrast to ADMA, SDMA remained unchanged and was related to serum creatinine level [37]. Our results are consistent with these data. All age groups of CD73−/− mice were characterized by elevated serum l-ornithine concentration in comparison to control. There was no significant difference in serum l-citrulline level between studied groups. However, we found significantly increased ratios of ornithine/arginine and ornithine/citrulline in all CD73−/− age groups in comparison to controls (Fig. 5). Kövamees et al. also demonstrated similar observations in patients with type 2 diabetes, which is one of the pro-inflammatory diseases, and macrovascular complications [38]. These observations are consistent with the enhanced activity of arginase (Fig. 4c), responsible for the hydrolysis of l-arginine to urea and l-ornithine, in relation to NOS. Such changes in enzymatic activities observed in CD73 mutant mice may be also related to decreased NO production and thus, vascular dysfunction [39].

Considering the lack of the CD73 activity on the erythrocytes (data not shown), the significantly lower AMP hydrolysis rate observed in the blood of CD73−/− mice compared to WT (Figure S2) was most likely resulted from the CD73 deletion on circulating cells such as lymphocytes. However, it is well known that intracellular nucleotide concentrations are on milimolar level and extracellular concentrations are much lower (nmolar–µmolar) [40, 41]. Therefore, the observed changes in blood adenine nucleotide concentrations were caused by intracellular processes. Metabolic conditions such as oxidative stress or inflammation could lead to an increase requirements for ATP or reduce its regeneration, thus decreasing the overall ATP bioavailability [42]. Despite the decrease in ATP and ADP concentrations as well as a reduced ATP/AMP ratio in CD73−/− mice blood due to the enhanced ATP degradation, no disturbed values of adenylate energy charge in CD73-deficient animals were noticed (0.93 ± 0.02 for WT; 0.90 ± 0.01 for CD73−/−). Moreover, ATP released from erythrocytes is an important regulator of synthesis by endothelial cells [43].

In conclusion, our study shows that the CD73 deletion leads to the development of age-dependent endothelial dysfunction in mice. Vascular damage is related to severe distrubances in l-arginine metabolism. The increase in CD73 activity could be protective in vascular pathologies.

## Electronic supplementary material

Below is the link to the electronic supplementary material.

Supplementary material 1 (TIFF 15978 kb)

Supplementary material 2 (TIFF 13231 kb)

Supplementary material 3 (DOCX 16238 kb)

Supplementary material 4 (TIFF 2028 kb)
