# Supplementary figures and images for: Impaired l-arginine metabolism marks endothelial dysfunction in CD73-deficient mice
Source: Mol Cell Biochem. 2019 May 15;458(1):133–42. doi: 10.1007/s11010-019-03537-4 (PMC6616215; doi:10.1007/s11010-019-03537-4)

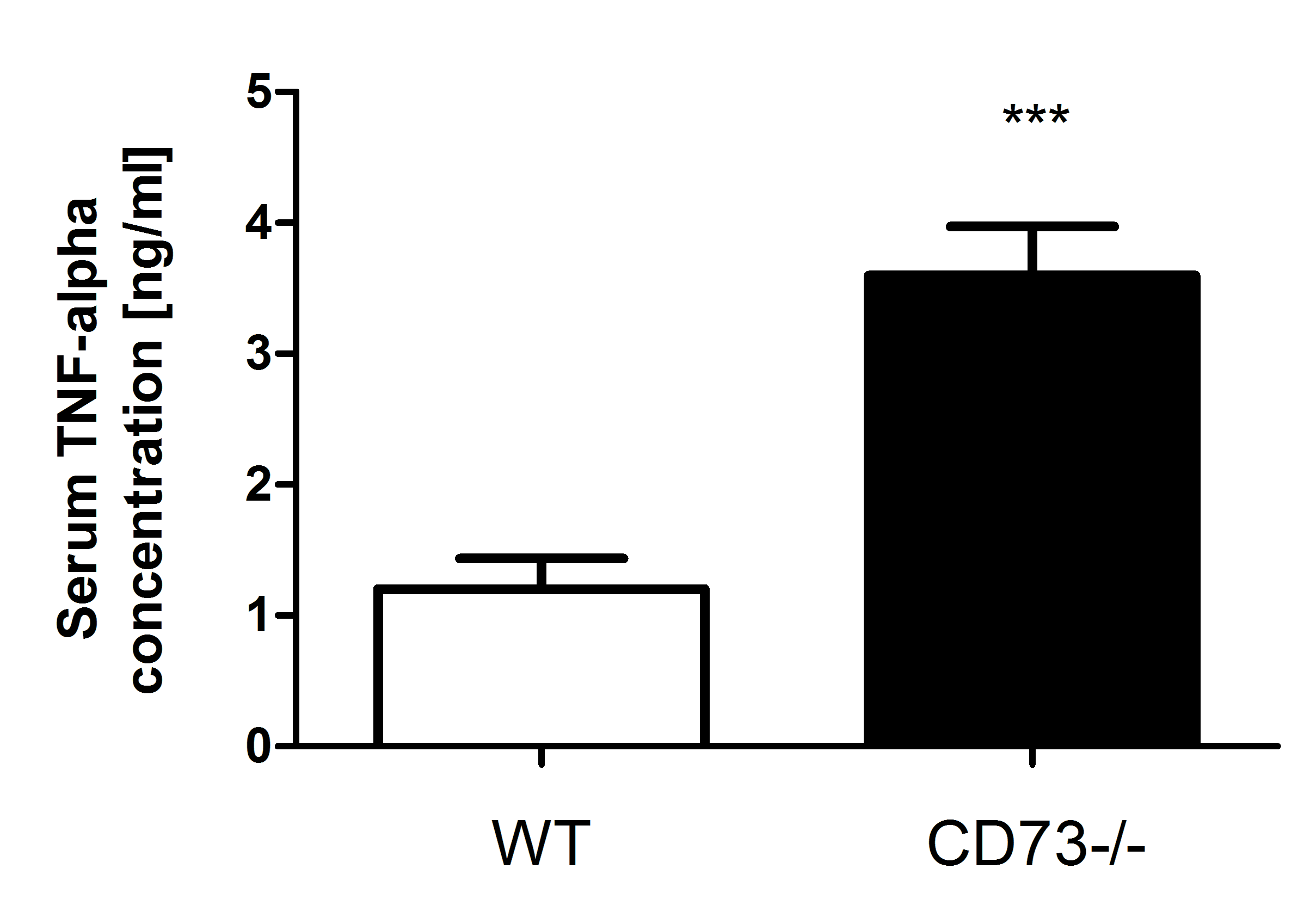

Supplement: Supplementary file 1 — Supplementary material 1 (TIFF 15978 kb) [file 11010_2019_3537_MOESM1_ESM.tif]

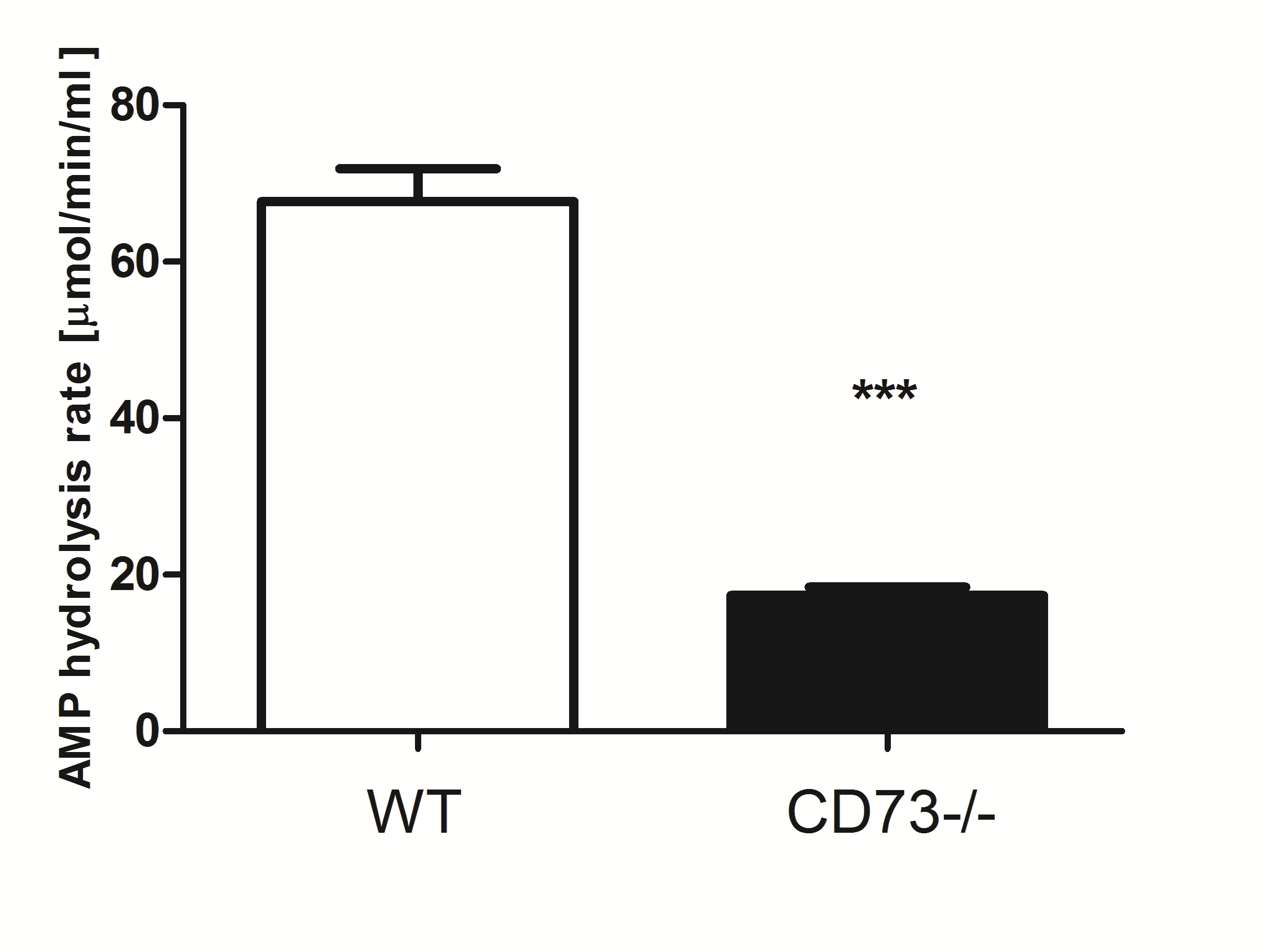

Supplement: Supplementary file 2 — Supplementary material 2 (TIFF 13231 kb) [file 11010_2019_3537_MOESM2_ESM.tif]

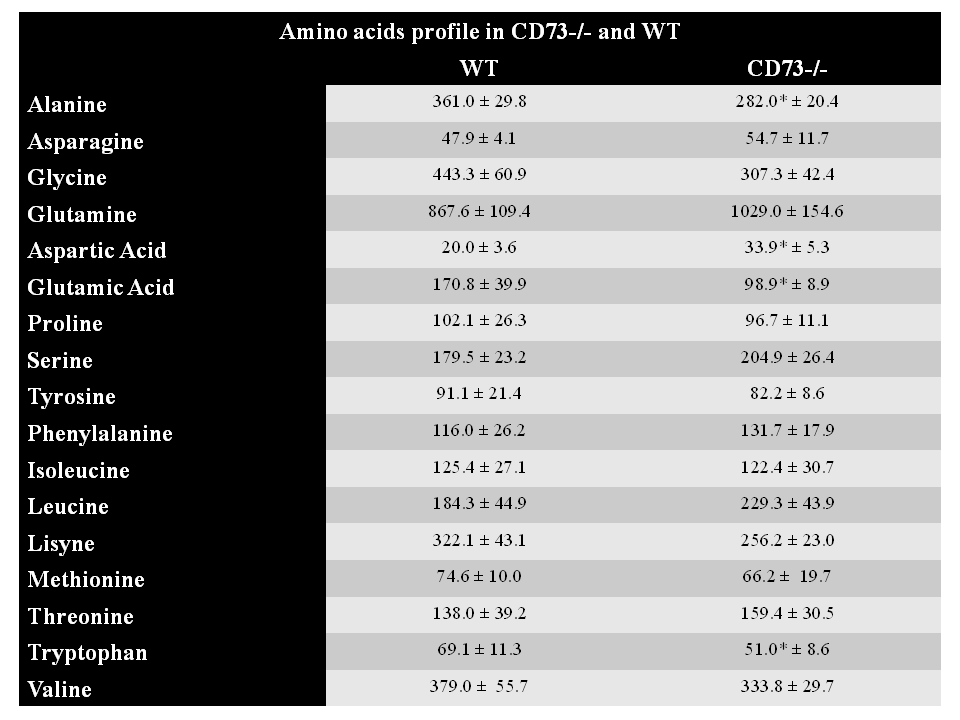

Supplement: Supplementary file 4 — Supplementary material 4 (TIFF 2028 kb) [file 11010_2019_3537_MOESM4_ESM.tif]
