# Supplementary material for: Impaired l-arginine metabolism marks endothelial dysfunction in CD73-deficient mice
Source: Mol Cell Biochem. 2019 May 15;458(1):133–42. doi: 10.1007/s11010-019-03537-4 (PMC6616215; doi:10.1007/s11010-019-03537-4)
Supplement: Supplementary file 3 — Supplementary material 3 (DOCX 16238 kb) [file 11010_2019_3537_MOESM3_ESM.docx]

Supporting Information

**Impaired L-arginine metabolism marks endothelial dysfunction in CD73-deficient mice**

Mierzejewska P^1^, Zabielska MA^1,2^_,_ Kutryb-Zajac B^1^, Tomczyk M^1^, Koszalka P^3^, Smolenski RT^1^, Slominska EM^1^

^1^ Department of Biochemistry, Medical University of Gdansk, Poland; ^2^ Department of Physiology, Medical University of Gdansk, Poland; ^3^ Department of Medical Biotechnology, Intercollegiate Faculty of Biotechnology UG-MUG, Medical University of Gdansk, Poland

**Corresponding Author:**

Dr Ewa M. Slominska,

Department of Biochemistry,

Medical University of Gdansk,

80-211 Gdansk, Debinki 1, Poland ,

Phone: +48 58 349 1464, Fax: +48 58 3491465,

e-mail: [eslom@gumed.edu.pl](mailto:eslom@gumed.edu.pl)

# MATERIALS AND METHODS

## Determination of blood AMP hydrolysis rate

Frozen whole blood samples from 6-month-old CD73-/- and WT mice were diluted 5 times with cold water. The samples were subjected to three cycles of thawing/ freezing at - 80º C. Then, blood lysates were diluted 5 times with CD73 assay buffer, containing 50 mM Tris-HCl (pH 8.0), 5 mM MgCl_2_, 10 mM B-glycerophosphate with 0.1 % Triton X-100 and 5 µM EHNA and incubated on ice for 1 hour. AMP hydrolysis rate was measured based on the increase of the product - adenosine after 15 - minute incubation of the diluted blood lysates at 37º C in the presence of 0.2 mM AMP. The reaction was stopped by addition of 1.3 M HClO_4_ followed by a centrifugation (20800 g/15 min/4 ºC). Supernatants were then collected and brought to pH 6.0–6.5 using 3 M K_3_PO_4_ solution. After 15 min incubation in ice to ensure KClO_4_ precipitation, samples were centrifugated at the same conditions (20800 g/15 min/4 ºC), and the supernatants were analysed using high performance liquid chromatography (HPLC) as previously described.

# SUPPLEMENTAL FIGURES


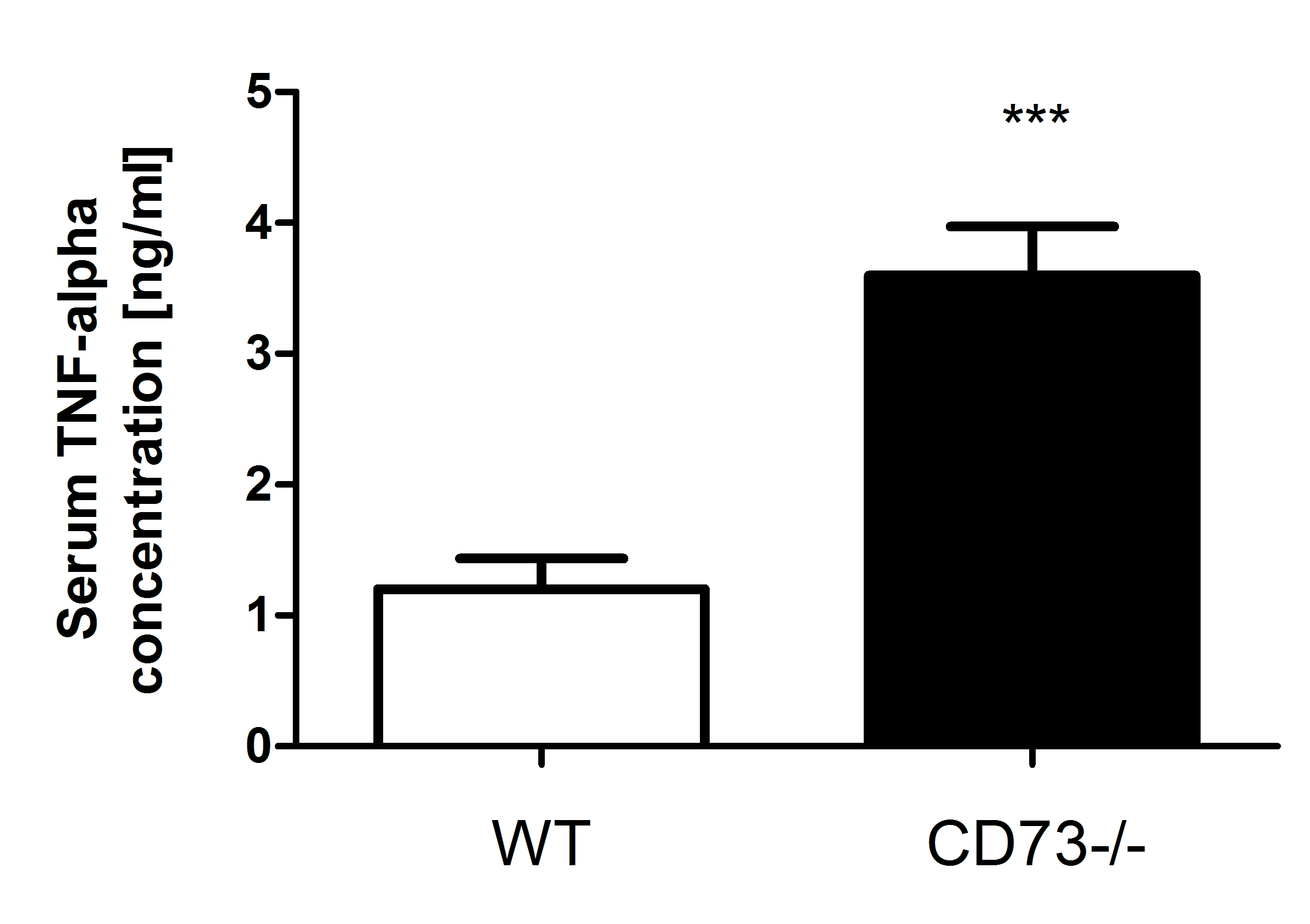


**Figure S1. Proinflammatory phenotype of CD73 - deficient mice, indicated by increased TNF-alpha level**. Serum TNF-alpha concentration of 6-month-old CD73-/- and WT mice. Values are shown as mean ± SEM (n=7, Student t test: *p<0.05; **p<0.01; ***p<0.001).


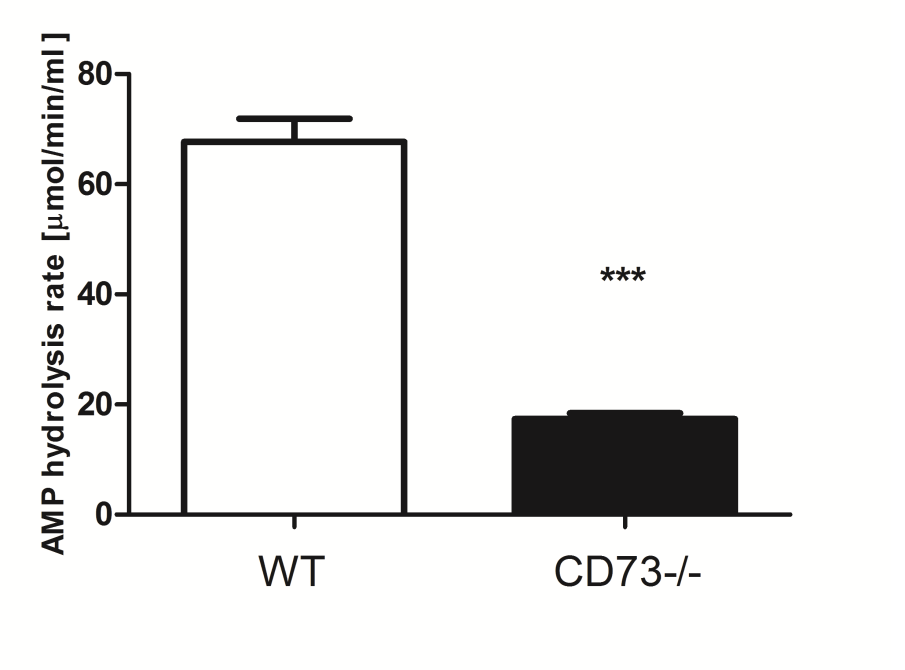


**Figure S2. CD73 deletion profoundly decreases the rate of AMP to adenosine conversion in blood.** AMP hydrolysis rate in 6-month-old CD73-/- and WT mice blood. Values are shown as mean ± SEM (n=7, Student t test: *p<0.05; **p<0.01; ***p<0.001).

**
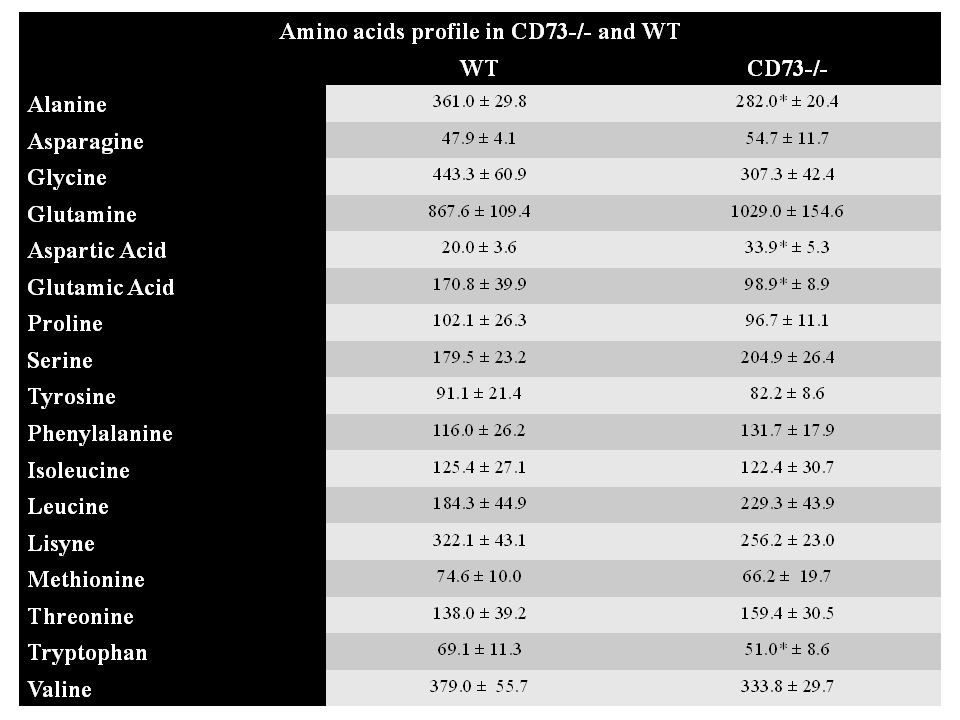
**

**Supplemental Table 1. Other amino acids levels in CD73-/- and WT mice serum.** Amino acids profile of 6-month old CD73-/- and WT mice. All values are shown as mean ± SEM, (n=5; Student t test: *p<0.05; **p<0.01; ***p<0.001).
